# Intratemporal Facial Nerve Schwannomas: A Review of 45 Cases in A Single Center

**DOI:** 10.3390/diagnostics12081789

**Published:** 2022-07-23

**Authors:** Tsubasa Kitama, Makoto Hosoya, Masaru Noguchi, Takanori Nishiyama, Takeshi Wakabayashi, Marie N. Shimanuki, Masaki Yazawa, Yasuhiro Inoue, Jin Kanzaki, Kaoru Ogawa, Naoki Oishi

**Affiliations:** 1 Department of Otorhinolaryngology-Head and Neck Surgery, Keio University School of Medicine, 35 Shinanomachi, Shinjuku-ku, Tokyo 160-8582, Japan; tsubasa.kitama@gmail.com (T.K.); mhosoya1985@gmail.com (M.H.); masaru_n0613@yahoo.co.jp (M.N.); tnmailster@gmail.com (T.N.); t12wakabayashi@yahoo.co.jp (T.W.); nukimari@gmail.com (M.N.S.); yas712hachiman@gmail.com (Y.I.); kanj1937@yahoo.co.jp (J.K.); ogawak@a5.keio.jp (K.O.); 2 Department of Plastic Surgery, Keio University School of Medicine, 35 Shinanomachi, Shinjuku-ku, Tokyo 160-8582, Japan; yazawa@a7.keio.jp

**Keywords:** facial nerve schwannomas, facial nerve palsy, temporal bone

## Abstract

There are no established indications for facial nerve schwannoma treatment, including surgery, radiation and follow-up observation, and it is difficult to determine treatment policy uniformly. The treatment policy was examined from each treatment course. Data of patients with facial nerve schwannomas at our hospital from 1987 to 2018 were retrospectively examined. Their age, sex, clinical symptoms, tumor localization, treatment policies and outcomes were reviewed. In total, 22 patients underwent surgery and 1 patient underwent radiotherapy; 22 patients were followed up without treatment. After total resection, there were no tumor recurrences, and most patients had grade 3 or 4 postoperative facial paralysis. After subtotal resection, tumor regrowth was observed in four patients and reoperation was required in two patients. Facial nerve function was maintained in four patients and was decreased in two patients. During follow-up, six patients showed tumor growth. Only one patient had worsening facial nerve paralysis; four patients underwent facial nerve decompression owing to facial nerve paralysis during follow-up. If the tumor compresses the brain or it is prone to growth, surgery may be indicated, and when the preoperative facial nerve function is grade ≤ 3, consideration should be given to preserving facial nerve function and subtotal resection should be indicated. If the preoperative facial nerve function is grade ≥ 3, total resection with nerve grafting is an option to prevent regrowth. If there is no brain compression or tumor growth, the follow-up is a good indication, and decompression should be considered in facial nerve paralysis cases.

## 1. Introduction

Facial nerve schwannoma is the most frequent tumor derived from the facial nerve [1]. It is benign and commonly grows slowly. It can occur at any location in the facial nerve, from near the brainstem to outside the temporal bone. The most common site of occurrence is in the temporal bone facial nerve canal, followed by the cerebellar pons, inner ear canal and parotid gland [2]. Previous studies have found sporadic facial nerve schwannoma in <1% of the temporal bone [3], but in clinical practice, it is often found during the close investigation of facial nerve paralysis. Facial nerve schwannoma may cause several other symptoms such as hearing loss, tinnitus, vertigo, cephalalgia and otalgia. When it increases and compresses the brain, it may cause neurological symptoms and signs such as paralysis and trigeminal neuralgia [4].

Although facial nerve schwannoma treatment mainly includes surgical intervention, radiation and follow-up observation, there are no established criteria for treatment selection [5]. Follow-up observation is an option for patients without facial paralysis and tumor growth. Total resection was the main surgical intervention until the 90s, but it has been recently limited to the tumors with progressive palsy or tumors that compress the brainstem, temporal lobe or cerebellum. Total resection requires nerve resection and reconstruction [2]. Subtotal resection has been a treatment option after the 2000s. Some reviews have shown that this operation enables nerve preservation by limiting the procedure to tumor debulking [6].

There are insufficient studies on the long-term course after subtotal resection. Facial nerve decompression can be performed when the tumor causes facial nerve paralysis during observation [7,8]. Some studies have reported the validity of radiation therapy, including gamma knife treatment. During the follow-up period of approximately 5 years, approximately 30% of the target cases require postoperative irradiation. The rate of good control is approximately 90%, and the facial nerve function improvement rate is 20%. In contrast, facial nerve function deterioration rates of 10–20% have been reported [9,10]. Most studies have evaluated tumors near the cistern, and there are limited studies on tumors in the temporal bone.

In facial schwannoma treatment, facial nerve function preservation has become increasingly important. Tumor regrowth after treatment should also be avoided as much as possible. There are many cases in which no increase or worsening of facial nerve paralysis is observed without treatment. Taken together, there are no established criteria regarding which case should be treated and which treatment option should be chosen. This study aimed to investigate the outcome of each treatment based on the largest number of cases in Japan to consider indications for each treatment.

## 2. Materials and Methods

The data of patients who were treated or observed at the Department of Otorhinolaryngology at Keio University Hospital from 1987 to 2018 (32 years) were retrospectively examined. This survey was approved by the Institutional Review Board of Keio University (Ethics Numbers 20140242, 20200033).

Their age, sex, clinical symptoms, tumor localization and treatment policies were reviewed. Tumor localization was classified into the cerebellopontine angle (CPA), internal auditory canal (IAC), geniculate ganglion (GG), horizontal portion of the facial nerve (H), vertical portion of the facial nerve (V) and parotid gland (PA).

## 3. Results

**Figure 1:**
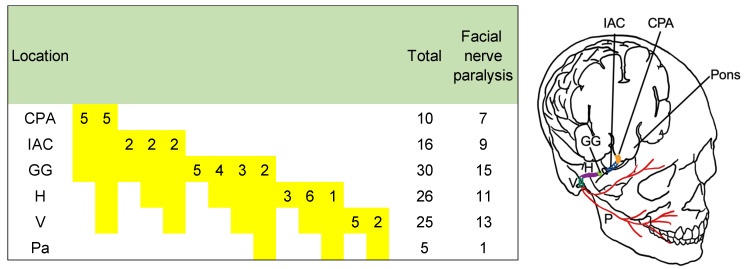
Tumor location and facial nerve function. CPA: cerebellopontine angle, IAC: internal auditory canal, GG: geniculate ganglion, H: horizontal portion of the facial nerve, V: vertical portion of the facial nerve, PA: parotid gland.

In total, 45 patients visited our outpatient department during the study period. There were 22 men and 23 women with a mean age of 43.9 years (13–74 years). Among the 23 patients with facial nerve paralysis, 16 had gradually advanced facial nerve paralysis, 4 had recurrent facial nerve paralysis and 3 had sudden-onset facial nerve paralysis. Among the 26 patients with hearing loss, 23 had conductive hearing loss, 7 had tinnitus, 4 had ear fullness and 2 had dizziness; 7 other patients had ear canal mass, 2 had otorrhea and 2 had parotid gland mass. Figure 1 shows the location of the tumor. Most tumors straddled >1 segment.

Regarding the location of the tumor, five tumors straddled the CPA, IAC and GG; five straddled the CPA, ICA, GG, H and V; two straddled the IAC and GG; two straddled the IAC, GG and H; two straddled the IAC, GG, H and V; six straddled the GG; four straddled the GG and H; three straddled the GG, H and V; two straddled the GG, H, V and Pa, three straddled the H; six straddled the H and V; one straddled the H, V and Pa; five straddled the V; and two straddled the V and PA. Among the 10 tumors extending to the CPA, 7 caused facial nerve paralysis. Among the 16 tumors extending to the IAC, 9 caused facial nerve paralysis. Among the 30 tumors extending to the GG, 16 caused facial nerve paralysis. Among the 26 tumors extending to the H, 11 caused facial nerve paralysis. Among the 25 tumors extending to the V, 13 caused facial nerve paralysis. Among the five tumors extending to the PA, one caused facial nerve paralysis.

The treatment policy was also examined and 22 patients eventually underwent operation; 16 underwent total resection and 6 underwent subtotal resection. One patient underwent radiotherapy and 22 patients were followed up without treatment; 4 of them underwent facial nerve decompression. Table 1 shows the features of patients treated with each treatment policy and Table 2 shows the outcomes of each treatment policy.

This table shows the patients’ features who underwent the following treatment: total resection, subtotal resection and follow-up. There is no tendency for age or sex in each treatment group, but it can be seen that there are many mild cases of facial nerve paralysis in the follow-up group and relatively many severe cases in the total resection group.

This table shows the outcomes of those who underwent the following treatment: total resection, subtotal resection and follow-up. Most facial nerve paralysis after total excision was grade 3 or 4 in the total-resection group. Many patients had preserved facial nerve function in the subtotal-resection group and the follow-up group. Tumors regrew in six patients during the observation. Among them, two patients underwent total resection, one underwent subtotal resection, two received radiation therapy and one was followed up.

The average age of patients who underwent total resection was 48.1 years (13–68 years), and there were eight men and eight women. Regarding preoperative facial nerve condition, nine patients were House–Brackmann grade [11] 1 and 2, four were grade 3 and 4, and three were grade 5. The amputated facial nerve was reconstructed with the hypoglossal nerve in eight cases and reconstructed with the nerve grafts of the auricular nerve or sural nerve in seven cases. There were no tumor recurrences after surgery. In most cases, postoperative facial paralysis was grade 3 or 4. Exceptionally, in three cases, the tumor derived from the large pyramidal nerve and the intermediate nerve was grade 1 or 2.

The average age of patients who underwent subtotal resection was 40.2 years (17–54 years), and regarding sex there were three males and three females. Regarding the preoperative facial nerve condition, four, one, one, and zero patients were classified as having House–Brackmann grades 1, 2, 3, and ≥4, respectively. The operation was performed via the middle fossa or transmastoid approach, and the tumor was removed using a nerve integrity monitor as much as possible. Tumor regrowth was noted in four patients after operation: two were completely resected and two were followed up. Postoperative facial nerve paralysis was grade 1 in three patients, grade 2 in two patients and grade 3 in one patient. In three patients, conductive hearing loss was observed before surgery; therefore, type IV reconstruction was performed, which improved hearing.

The average age of the initially followed-up patients was 44.1 years (16–74 years), and there were 10 men and 12 women. Regarding the facial nerve condition at the first visit, 13, 2, 2, 3, and 2 patients were classified as having grades 1, 2, 3, 4, and 5, respectively. Tumors grew in six patients during the observation. Among them, four patients showed tumor growth in the CPA, of which two patients underwent total excision, one underwent radiation therapy and one was followed up. Further, two patients showed tumor growth in the GG: one patient underwent debulking surgery and the other patient received radiation therapy. Tumors in the H or V showed little growth. During observation, 2 patients had worsening facial nerve paralysis, and the remaining 20 had preserved facial nerve function.

Four patients underwent facial nerve decompression. Their average age was 30.8 years (24–40 years), and there were two men and two women. Regarding the facial nerve condition at the first visit, one, one, one, and one patient was classified as having grades 1, 3, 4, and 5, respectively. The duration of paralysis was <3 weeks in two patients, who were classified as having grades 3 and 5; approximately 2 months in one patient, who was classified as having grade 4; and >1 year (chronic) in the remaining one patient, who was classified as having grade 2. Only one patient with facial nerve paralysis recovered after operation (from grade 5 to grade 1). In the recovered patient, the tumor was in the vertical portion, and the preoperative electroneurography value was 0%. The operation was performed 3 weeks after the onset.

## 4. Discussion

Treatment for facial schwannoma has changed over time, and because it is a rare tumor, there are various debates regarding the treatment options [12]. Tumors do not often regrow even after subtotal resection, and facial nerve paralysis greatly impairs the quality of life. Malignant tumor transformation is extremely rare, and diagnostic imaging of the tumor by CT and MRI becomes accurate. There is a tendency to prioritize the preservation of facial nerve function over total tumor removal. In this study, the data of patients who were followed up or treated at our department were retrospectively considered to examine treatment strategies.

In this study, 22 patients were initially followed up. In all patients, the tumors did not compress the brain, and most patients had maintained facial nerve function of House–Brackmann grade 1 or 2. In some patients, even if facial nerve paralysis was grade ≥ 3, the tumors that did not show a tendency to grow were followed up with periodic imaging. Less than 30% of the tumors grew during follow-up and only one patient had worsened facial nerve function. Preservation of facial nerve function should be prioritized generally, and conservative follow-up is the first choice rather than interventional treatment such as surgery and radiation therapy. If there is no brain compression and no facial nerve paralysis of House–Brackmann grade ≥ 3, follow-up should be performed first. Some reports show that the average annual growth of the tumor is approximately 1–2 mm [5], and larger tumors are more likely to cause subsequent growth [13]. Follow-up with careful imaging is essential. If facial nerve paralysis occurs during follow-up, steroids should be administered first. Facial nerve decompression should be considered, especially for intratemporal tumors in an early stage [8]. In this study, among the four patients with decompression, one recovered and the condition of three did not worsen.

Surgery for schwannoma can be divided into total or subtotal resection. In this study, 16 patients underwent total resection. Regarding total resection, there are many cases in which the tumor and nerve are unclearly marginated, and nerve fibers are found almost all around the tumor, especially in the temporal bone. Thus, total excision of the tumor in the temporal bone or brain cistern is not usually able to avoid facial nerve paralysis. Before 2000s, total tumor resection was performed for almost all cases [7,14]. When total resection with nerve grafting is achieved, good long-term tumor control and facial nerve function of House–Brackmann grade 3–4 are expected. In this study, facial nerve function was not preserved after surgery in all patients without tumor recurrence except cases with tumors originated from the large pyramidal nerve or the intermediate nerve, and most facial nerve paralysis after total excision was grade 3 or 4. Liu et al. reported that facial nerve paralysis after total resection was almost House–Brackmann grade 3 [15]. If the preoperative House–Brackmann grade is ≥3, total tumor resection and nerve transplant reconstruction are indicated. Similar opinions have been expressed in previous literature [16,17]. The facial nerve function after reconstruction improves as the period of facial nerve paralysis before surgery becomes shorter. Surgery should be considered as soon as possible once facial nerve paralysis has progressed to grade 3 or 4 [2,18].

Although there are insufficient studies on the long-term course after subtotal resection, some studies have shown good nerve-sparing rates after resection for tumors from the cerebellar pontine corner to the inner ear canal [6,19]. In six patients included in this study, four achieved preservation of the facial nerve function. Deterioration of the facial nerve function was observed in two patients but it was very mild. Further, four of six (66.7%) patients had tumor regrowth, and two patients finally required reoperation. Previous literature also reported a recurrence rate of 26.7% [20], and there is a risk of regrowth with subtotal resection. Previously, nerve monitoring using a nerve integrity monitor was used. Recently, continuous monitoring of facial nerves using the facial nerve root exit-zone-elicited compound muscle action potential has become possible [21,22,23], and there is a possibility that the nerve preservation rate can be further increased in the future. It may be an effective option for growing tumors if patients are relatively young and have good facial nerve function. There is also a treatment algorithm that positions subtotal resection as a treatment option for intracranial tumors measuring >3 cm that maintain good facial nerve function [24].

Regarding the surgical method, various surgical methods, such as the suboccipital craniotomy approach, the middle cranial fossa approach, the translabyrinthine approach, the approach that combines the middle cranial fossa with transmastoid, the transmastoid approach, excision according to the parotid tumor and temporal bone surgery with parotid gland surgery, are used depending on the location of the tumor, degree of residual hearing and experience of the operator [2]. It is necessary to be familiar with various approaches in each area of temporal bone surgery, head and neck surgery, neurosurgery and plastic surgery. Surgical treatment in an experienced facility that can provide team medical care by multiple departments is desirable.

For parotid gland tumors, it is necessary to consider the natural course, as facial nerve paralysis rarely occurs during the follow-up period. A treatment algorithm has been proposed according to whether the tumor extends intratemporally [25]. In the case of intratemporal extension, follow-up after decompression is recommended. In the case without intratemporal extension and if the tumor can be detached from the nerve or if the tumor occurs in a peripheral branch rather than the main trunk, total resection with nerve grafting is recommended. If detachment from the nerve is difficult, follow-up is recommended.

Radiation therapy is a good treatment option for patients with a tendency for tumor growth but with good facial nerve function, such as House–Brackmann grade 1–3 [26]. The treatment outcomes for tumors located more centrally from the geniculate ganglion have been reported. The treatment outcomes for tumors in the temporal bone are unknown [9]. Radiation therapy has not been confirmed to be effective for facial nerve schwannoma of the temporal bone and is not an active indication at the moment.

Facial nerve paralysis is the most important symptom of facial nerve schwannoma, which especially affects quality of life, and it has been examined from the same viewpoint in the past [2,7,8,27]. Thus, the treatment indication was examined from facial nerve paralysis and tumor growth mainly in this study. Facial nerve schwannoma also causes hearing loss, dysgeusia and ocular symptoms, so it may be necessary to consider the treatment policy from such a viewpoint in addition.

## 5. Conclusions

**Figure 2:**
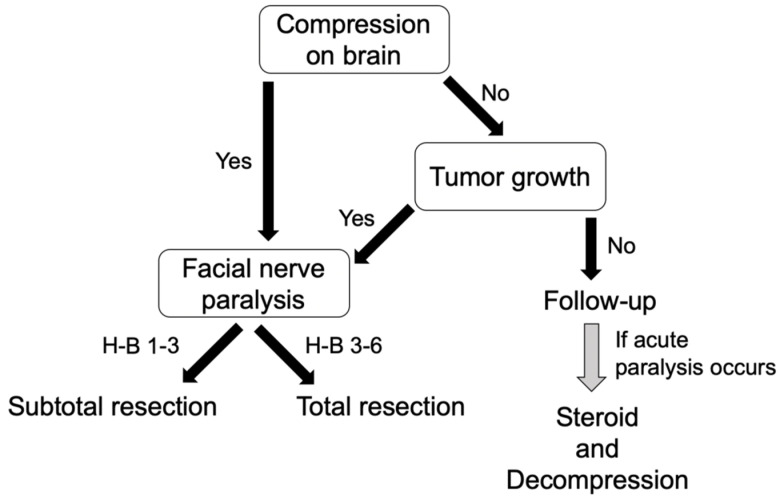
Treatment policies for intratemporal facial nerve schwannoma.

Figure 2 shows the treatment strategies for intratemporal facial nerve schwannoma. If the tumor compresses the brain or if the tumor is prone to growth, surgery may be indicated, and when the preoperative facial nerve function is grade ≤ 3, consideration should be given to preserving facial nerve function, and subtotal resection should be indicated. If it is grade ≥ 3, total resection with nerve grafting is an option to prevent regrowth. If there is no brain compression or tumor growth, follow-up is a good indication, and when facial paralysis appears, decompression should be considered.

Treatment strategies for intratemporal facial nerve schwannoma are first divided according to whether the tumor is compressing the brain. If the brain is compressed, or if tumor is prone to grow, surgical treatment is indicated. If the facial nerve paralysis is H-B grade 1–3, subtotal resection is required, and if H-B grade is 3–6, total resection is required. If no compression of the brain and no tumor growth is observed, patients should be followed up. If acute facial paralysis develops during follow-up, decompression should be considered.

## Figures and Tables

**Table 1:** Features of patients with each treatment policy.

		Total Resection (n = 16)	Subtotal Resection (n = 6)	Follow-up (n = 22)
	Age (Mean ± Standard Deviation)	48.1 ± 16.7	40.2 ± 12.5	44.1 ± 17.4
Sex	Male	8	3	10
	Female	8	3	12
Facial nerve paralysis	Grade 1, 2	9	5	15
	Grade 3, 4	4	1	5
	Grade 5, 6	3	0	2

**Table 2:** Outcomes of each treatment policy.

	Total Resection (n = 16)	Subtotal Resection (n = 6)	Follow-up (n = 22)
HB grade at diagnosis			
1	5	4	13
2	4	1	2
3	3	1	2
4	1	0	3
5	3	0	2
6	0	0	0
HB grade at last follow-up			
1	1	3	14
2	2	2	3
3	5	1	2
4	5	0	2
5	3	0	1
6	0	0	0
Change in HB grade			
Same or improved	7	4	20
Worse	9	2	2
Cases in which the treament policy was changed during follow-up			
total resection		2	
subtotal resection		1	
radiation therapy		2	

## Data Availability

The datasets generated and analyzed during the current study are available from the corresponding author on reasonable request.
